# Genome-Wide Identification, Characterization, and Expression Analysis of the MYB-R2R3 Gene Family in Black Pepper (*Piper nigrum* L.)

**DOI:** 10.3390/ijms25189851

**Published:** 2024-09-12

**Authors:** Rui Fan, Kai Huang, Zhican Zhao, Yupeng Hao, Xueying Guan, Haiyan Luo, Chaoyun Hao

**Affiliations:** 1 Spice and Beverage Research Institute, Chinese Academy of Tropical Agricultural Science (CATAS), Wanning 571533, China; fanrui_83@catas.cn; 2 China Zhejiang Provincial Key Laboratory of Crop Genetic Resources, Institute of Crop Science, Plant Precision Breeding Academy, College of Agriculture and Biotechnology, Zhejiang University, Hangzhou 310058, China; 22316175@zju.edu.cn (K.H.); 22016137@zju.edu.cn (Y.H.); 3 Hainan Institute of Zhejiang University, Building 11, Yonyou Industrial Park, Yazhou Bay Science and Technology City, Yazhou District, Sanya 572025, China; xueyingguan@zju.edu.cn; 4 College of Tropical Crops, Yunnan Agricultural University, Pu’er 665099, China; 2020314993@stu.ynau.edu.cn; 5 Tropical Croups Genetic Resources, Chinese Academy of Tropical Agricultural Science (CATAS), Haikou 571101, China; haiyan2022@catas.cn

**Keywords:** black pepper, R2R3-MYB transcription factors, *Phytophthora capsici*, gene expression

## Abstract

Black pepper (*Piper nigrum* L.), a prominent spice crop, known as the “king of spices”, originated from India. The growth and development of black pepper are influenced by various environmental conditions. MYB transcription factors play a crucial role in controlling metabolic processes, abiotic stress management, and plant growth and development. In this study, we identified 160 *PnMYB* transcription factors in the black pepper genome. Phylogenetic analysis was performed using 125 R2R3-MYB proteins from black pepper and *Arabidopsis thaliana*, resulting in the mapping of 20 groups on the phylogenetic tree, each containing members from both species. Most members of the *PnMYB* family possess two introns, and motif 3 and motif 4 are conserved in all members. The number of genes on each chromosome ranges from 1 to 10. Collinear analysis indicated the creation of new members through gene fragments and tandem replication. The Ka/Ks ratio indicated that purifying selection and positive selection acted on *PnMYB* of pepper. The majority of pepper *PnMYB* family members were in the nucleus. Significant differences in gene expression levels were observed between different species and infection periods when *Piper nigrum* L. and *Piper flaviflorum* were infected with *Phytophthora capsici*. These findings are valuable for future studies on the biological role and molecular mechanism of the *PnMYB* gene.

## 1. Introduction

Black pepper (*Piper nigrum* L.), also known as the “king of spices”, is native to South India and thrives in tropical regions. It is an important spice crop with a focus on export in most of the producing countries [1,2]. However, black pepper productivity is severely limited by pests and diseases, particularly by the infection of *Phytophthora capsici*, a mycotic pathogen that causes foot rot [3]. Vulnerable to *Phytophthora capsici* during all growth phases, pathogen infection of black pepper occurs via soil transmission to roots or through the aerial phase, which infects the leaves, stems, and panicles [4,5]. In order to defend against pathogen invasion, plants have developed a signaling network that activates a series of resistance genes. In *Piper colubrinum*, a wild relative of black pepper, the R (resistance) gene was found to be expressed without requiring induction by pathogenic bacteria and to be constitutively expressed with an upregulated expression at the onset of *P. capsici* infection [6]. In addition, the *CaSBP11* and *CaSBP12* genes from the SBP-box gene family function similarly to MYB and suppress the defense response against *P. capsici* [7,8]. The MYB transcription factors *MYB2R4*, *MYB2R3*, and *MYB3R6* have been shown to regulate different aspects of sporulation in Phytophthora oomycetes (*Phytophthora latina*) [9]. Upon infection with *P. capsici*, the expression patterns of *PR-2* (β-1,3-glucanase), *PR-5* (osmotin), and *PR-9* (cytoplasmic ascorbate peroxidase) in the resistant black pepper line exhibit significant up-regulation, and in the susceptible line, they are expressed maximally the first day after inoculation (DAI) but thereafter undergo down-regulation [10].

MYB transcription factors belong to one of the largest families of transcription factors and are characterized by the presence of N-terminal MYB DNA-binding domain (DBD) [11]. This domain consists of one to four imperfect repeats (Rs), with each hosting approximately 52 amino acid residues folded into three α-helices. The second and third helices of each repeat can form a helix-turn-helix (HTH) structure, and the third helix is the “recognition helix” that intercalates in the major groove of target gene DNA molecule [12]. Plant MYB proteins are divided into four subfamilies based on the number and location of MYB domains: “MYB-related genes (1R-MYB)”, “MYB-R2R3 (2R-MYB)”, “MYB-R1R2R3 (3R-MYB)”, and “Atypical MYB genes (4R-MYB)” [13]. The MYB-R2R3 subfamily, which contains two MYB repeats, is the largest subfamily of plant MYB genes. R2R3-MYB transcription factors are reported to involved in the regulation of plant-specific processes, including secondary metabolism, development, cell fate and identity, response to biotic and abiotic stresses [14]. For example, the R2R3-MYB transcription factor *OsMYBAS1* has been shown to improve seed germination under deep sowing conditions of rice [15], and the inhibition of SmMYB52 in Salvia miltiorrhiza has been found to suppress root growth and indole-3-acetic acid (IAA) accumulation [16]. MYB genes also play a role in the regulation of anthocyanin synthesis and abiotic stress responses in plants [13,17,18,19,20,21,22].

An increasing number of studies demonstrated that MYB transcriptions play a pivotal role in plant disease resistance. It has been shown that most of the MYB transcription factors involved in regulating plant disease resistance belong to the R2R3-MYB family [23]. *OsJAMyb* belongs to the R2R3-type MYB transcription factor, and overexpression of *OsJAMyb* in rice has been shown to enhance blast resistance in transgenic rice [24]. In eggplant, MYB44 acts as a positive regulator to activate the expression of spermidine synthase and improves the tolerance of eggplant to bacterial wilt [25]. *TaPIMP2*, a R2R3-type MYB gene, positively regulates resistance to sharp eyespot in wheat [26]. The expression of some mYB genes involved in defense responses is induced after pathogen infection. In transgenic tobacco, the expression of tomato *SpMYB* is significantly induced after *Fusarium oxysporum* and *Botrytis cinerea* infection [27]. In wheat, *TaMYB391* can be induced to participate in the resistance response to stripe rust caused by *Puccinia striiformis* f. sp. *tritici* infection [28]. The transcription factor encoded by the multiple-disease resistance (MDR) gene *ZmMM1* in maize contains a MYB structural domain that positively regulates related resistance genes, resulting in greater disease resistance in maize [29].

Genome-wide identification and analysis of MYB gene families have been conducted in various plants, such as mango, camphor, cacao, wheat, rice, grapevine, tomato, and cucumber, to uncover new gene functions and provide a foundation for future research [17,21,28,30,31,32,33]. However, only a few MYB proteins have been shown to play a role in stress tolerance in pepper. In this study, we aimed to comprehensively understand the *PnMYB* gene and investigate its role in the growth of black pepper plant, as well as the transcriptional regulation mechanism of the MYB gene family in pepper following fungal invasion. Genome-wide identification, characterization, and expression analysis of the MYB gene family were performed to provide a theoretical framework for future research. We compared the expression levels of MYB transcription factors in *P. flaviflorum* and *P. nigrum* at different time points after *P. capsici* infection. The crucial role of *PnMYBs* in modulating black pepper infection has implications for the development of bacterial and fungal antagonists for biological control [5].

## 2. Results

### 2.1. Identification and Physicochemical Properties Analysis of Black Pepper MYB

The 125 *Arabidopsis* MYB protein sequences collected from TAIR (http://www.arabidopsis.org/ (accessed on 16 September 2022)) were used as query sequences for the *P. nigrum* proteome database to retrieve the MYB family genes by eliminating redundant entries in the black pepper genome database. A total of 160 R2R3-MYB genes with PF00249, PF11831, PF13921, and PF14379 domains were identified and labeled from *PnMYB1* through to *PnMYB160* (Supplementary Table S3). The peptide sequences of *PnMYBs* ranged in length from 101 (*PnMYB155*) to 1129 (*PnMYB98*) amino acids (aa) with an average length of 333 aa, the isoelectric points ranged from 4.83 to 11.33, and the aliphatic index was between 52.28 and 91.74. The molecular weights also varied widely, ranging from 11,708.52 Da (*PnMYB155*) to 121,820.3 Da (*PnMYB98*). The grand mean of hydropathicity results showed that the hydrophilic proteins accounted for 100% of the total. Furthermore, all but three PnMYB proteins (*PnMYB16*, *PnMYB160*, and *PnMYB45*) had instability indices greater than 40, suggesting the majority of PnMYB proteins are potentially unstable (Supplementary Table S4).

### 2.2. Multiple Sequence Alignment and Phylogenetic Analysis

**Figure 1:**
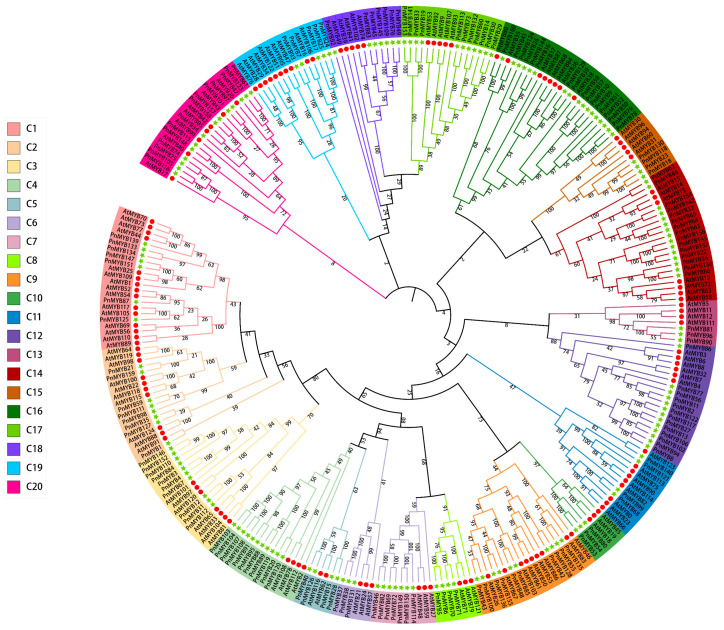
Phylogenetic tree of *PnMYBs*. At represents *A. thaliana*; Pn represents *P. nigrum*. The unrooted NJ tree was constructed based on MYB protein sequences from black pepper and *A. thaliana* using the MEGA10. Group names are on the outer ring and different groups are shown in different colors. All *PnMYBs* with their *A. thaliana* homologues were classified into 20 groups. Proteins from black pepper and *Arabidopsis* are denoted by green pentagrams and red circles.

To further investigate the differences among the MYB proteins, multiple sequence alignment was performed. The analyses showed that conserved tryptophan residues in the R2R3-MYB MYB domains of pepper, with slight variations in the first tryptophan of R3 (Supplementary Figure S1). To explore the evolutionary relationships of the MYB family, a phylogenetic tree was constructed using the MYB protein sequences from *A. thaliana* and *P. nigrum*. MYB was divided into 20 paraphyletic groups (Groups 1 to 20 or C1 to C20) (Figure 1). The number of *PnMYBs* was not equal in each group. The largest group, Group 16, contained 17 *PnMYBs* (10.625% of the total), while the lowest number of *PnMYBs* was in Groups 5, 6, and 10, with only 6 *PnMYBs* (1.875% each). Of these 20 concatenated groups, 4 had high bootstrap values. The presence of *AtMYBs* and *PnMYBs* in each group indicates a close relationship between black pepper and *A. thaliana*.

### 2.3. Gene Structure, Motif Patterns, and Chromosome Location of MYBs

**Figure 2:**
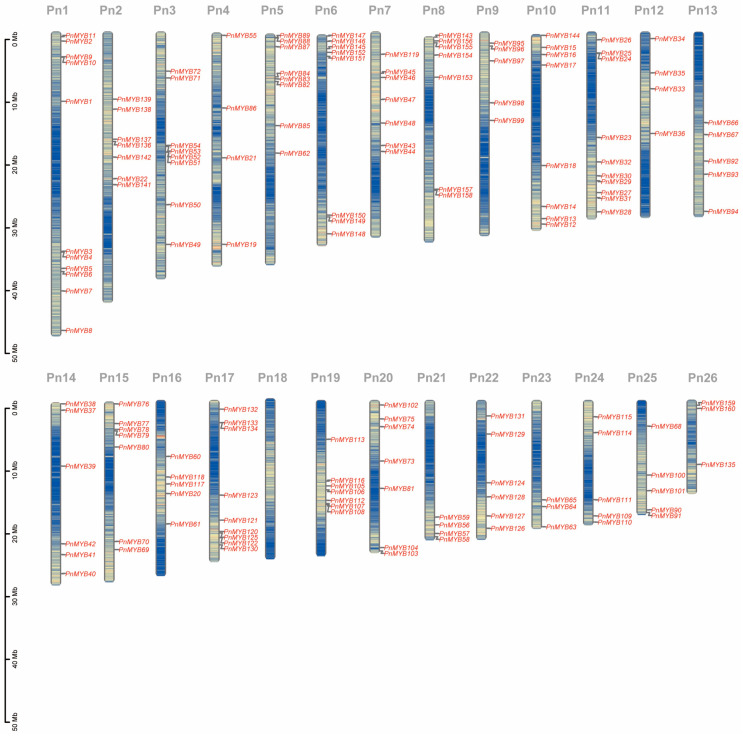
Chromosome location of *PnMYBs*. A total of 160 *PnMYBs* were found to be distributed on 26 chromosomes. The chromosome names are demonstrated at the top of each chromosome and the left-side scale is in megabases (Mb).

The chromosomal locations of *PnMYB* genes were mapped, and a total of 160 *PnMYBs* were found to be distributed on 26 chromosomes (Figure 2). The distribution of these genes varied across the different chromosomes. Pn1 contained the most genes, with 11 members. By contrast, Pn23 and Pn26 harbored the fewest genes, only three each. There were no MYB genes on Pn18. Eight *PnMYBs* were present on Pn3, Pn5, Pn6, and Pn10; seven on each of Pn2, Pn7, Pn8, Pn15, Pn19, and Pn20. We also found some *PnMYB* genes cluster in the black pepper genome. The presence of two or more gene clusters on chromosomes 1, 5, 8, 11, 17, and 19 suggests the possible existence of tandem duplication events. In addition, some of the genes were located at the end of chromosomes. For example, *PnMYB147* is located at the top of chromosome 6 and *PnMYB103* at the bottom of chromosome 20.

To explore the diversity of *PnMYB* gene structures, we analyzed the distribution of exon–intron structures. The analysis revealed that the exon–intron structure of *PnMYB* varied in number and length. The majority (73.75%) of *PnMYB* genes contained two introns, while three genes (*PnMYB139*, *PnMYB133*, and *PnMYB134*) lacked introns (Supplementary Figure S2). Four (2.5%) *PnMYB* genes contained more than 10 introns, of which *PnMYB98* had the most, up to 15, followed by *PnMYB136*. Similar patterns of intron and exon distribution were observed for genes within groups 6, 8, 11, 15, 19, and 20.

To further reveal the structural diversity and functional characteristics of MYBs, the motif patterns among *PnMYBs* were investigated. Motif analysis identified a total of fifteen motifs, with the number of conserved motifs ranging from two to eight, of which six highly conserved motifs are present in all *PnMYBs* (Supplementary Figure S3). Similarly, motifs 3 and 4 were detected in all *PnMYBs*, and more than 95% of *PnMYB* genes contained motifs 1 and 2. In addition, the functional differences in *PnMYB* genes may be due to the specific distribution of motifs, with nine motifs exclusively identified in specific *PnMYB* groups. Combined with phylogenetic tree analysis, it was found that motif 11 was solely present in Group 7, motif 14 was only existed in Group 14, and motif 10 was unique to Groups 14 and 16. Despite several unique motifs were found between groups, *PnMYBs* within the same group typically exhibited analogous motif patterns, such as *PnMYB103* and *PnMYB104*, as well as *PnMYB10* and *PnMYB58*, thereby implying that genes with corresponding positions in the phylogenetic tree might possess extremely similar functionalities.

### 2.4. Collinearity Analysis of the MYB Family in Black Pepper

**Figure 3:**
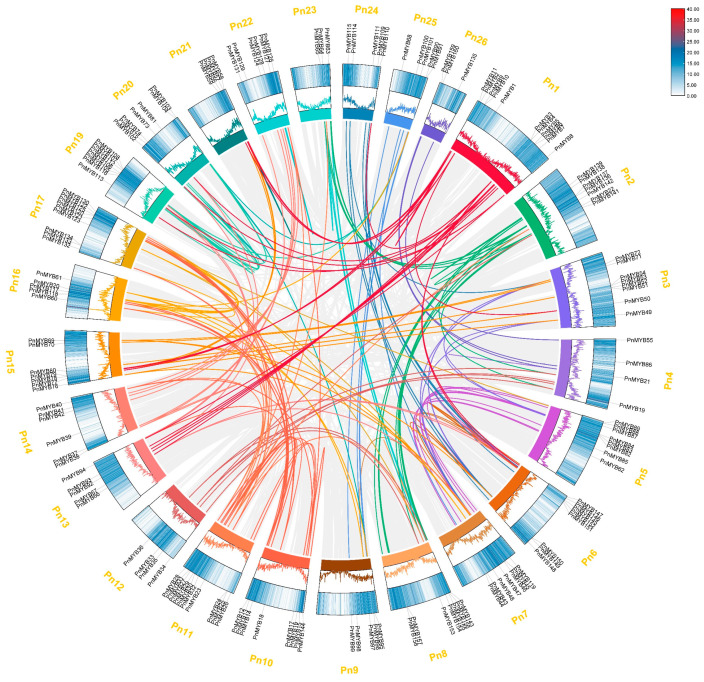
Chromosomal distribution of *PnMYB* genes in black pepper. Ribbon links indicate segmental duplication events between genes. Chromosome numbers are indicated outside in yellow. The gene names on each chromosome are indicated in the outer circle.

In order to examine the collinearity of the MYB gene family in the black pepper genome, we conducted a collinearity analysis of the black pepper MYB family genes. A total of 205 gene pairs exhibited collinearity in 26 chromosomes of the black pepper genome (Figure 3 and Supplementary Table S5). This suggests that a fundamental role for segmental duplications in differentiating *PnMYB* genes within the black pepper genome. All identified segmental duplication gene pairs were distributed on different chromosomes of the black pepper genome, and each chromosome contained a distinct number of gene pairs, indicating variations in collinearity patterns. To assess selective pressure on the *PnMYB* protein-coding gene, we calculated the Ka/Ks ratios of tandem and segmental duplication gene pairs. The analysis showed that only two pairs of fragment repeats showed Ka/Ks > 1, ranging from 1.01 to 2.84. The rest had 192 pairs of fragment repeats displaying Ka/Ks < 1, ranging from 0.00 to 0.93. The results showed that *PnMYBs* were affected by both purifying selection and positive selection, with purifying selection being more dominant.

### 2.5. Subcellular Localization of PnMYB Family

The subcellular localization of the MYB family members in pepper was investigated. Subcellular localization analysis revealed 18.125% of the MYB family members exhibit extracellular localization, while 0.625% resided in the chloroplast and a significant 81.25% were localized in the nucleus (Supplementary Table S6). Notably, the majority of *PnMYBs* are nuclear-targeting proteins, and previous studies have reported the nuclear localization of diverse MYB transcription factors, such as *AtMYB30*, *TaMYB391*, *TaMYB80*, *OsMYBAS1*, *TaPIMP2,* and *TaRIM1* [15,28,34].

### 2.6. Expression Pattern Analysis of PnMYB Gene Family

**Figure 4:**
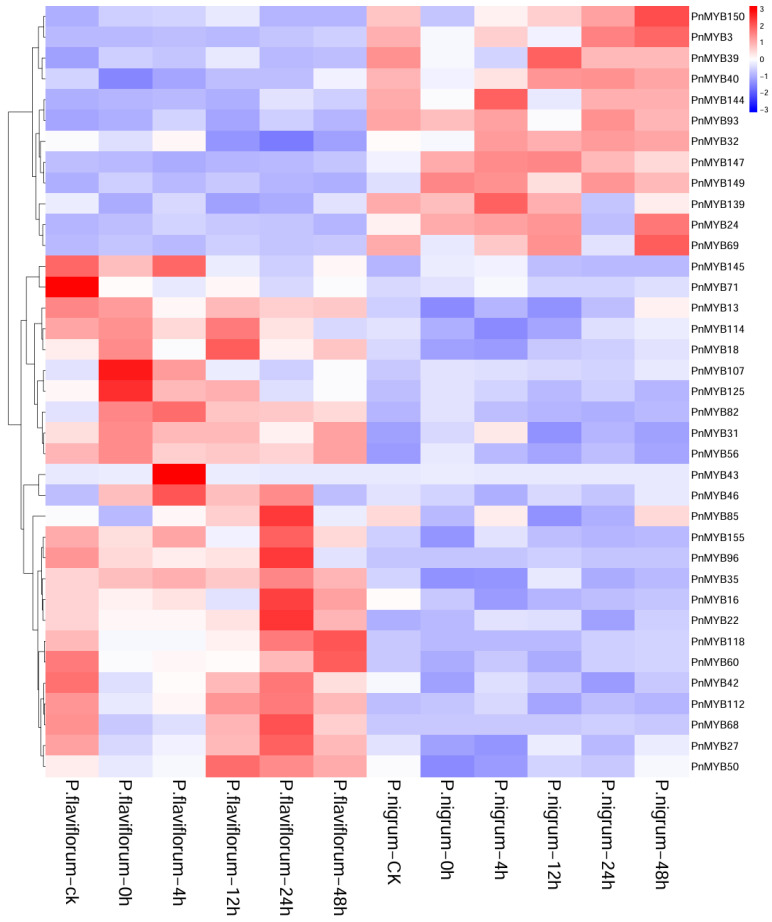
Expression profiles of 36 genes in the *PnMYB* family. Expression profiles of *PnMYB* genes in plants inoculated with *P. capsici* and in CK plants. Colors from blue to red represent the range of relative expression levels from low to high, respectively.

*Piper flaviflorum*, a close relative of black pepper, is resistant to *P. capsici* [35], in contrast to the susceptible cultivar black pepper. To examine the expression patterns of the MYB gene family in pepper, the RNA-seq data from two Piper plants *P. flaviflorum* and *P. nigrum* triggered by *P. capsici* were used to select differentially expressed genes. Thirty-seven MYB genes were selected and the RNA gene expression patterns of two species of Piper infected by *P. capsici* were analyzed at various time points. Our analysis revealed similar expression levels within the same phylogenetic taxa. For example, *PnMYB150* and *PnMYB3*, as well as *PnMYB50* and *PnMYB27,* originate from disparate phylogenetic taxa. Among these, the former pair exhibited consistently low expression levels throughout each stage in *P. flaviflorum*, while the latter pair maintained low expression at each stage in *P. nigrum*. Furthermore, we noted significant differences in the expression levels of the screened genes between different types, irrespective of whether uninfected or subjected to infection at diverse times. For instance, in *P. flaviflorum*, the expression levels of *PnMYB107* and *PnMYB125* rapidly increased from −1 and 0 to 3 at 0 h post infection, whereas the expression levels of *PnMYB43* and *PnMYB46* showed quick rises from −1 and −2 to 3 and 2 at 4 h following the infection. In contrast, the expression level of *PnMYB71* was significantly lower at each stage after exposure to *P. capsici*, which is a trait not observed in *P. nigrum*. Additionally, 12 h following infection with *P. capsici*, the expression level of *PnMYB85* in *P. nigrum* dropped from 2 to −3 but then rapidly increased to baseline level 48 h thereafter. Among all the genes assessed, *PnMYB43* was only highly expressed at 4 h after *P. flaviflorum* infection with *P. capsici*, with the expression level remaining low at other times. The heat map of gene expression is presented in Figure 4.

### 2.7. Expression Differences in MYB Genes in Two Piper Species

**Figure 5:**
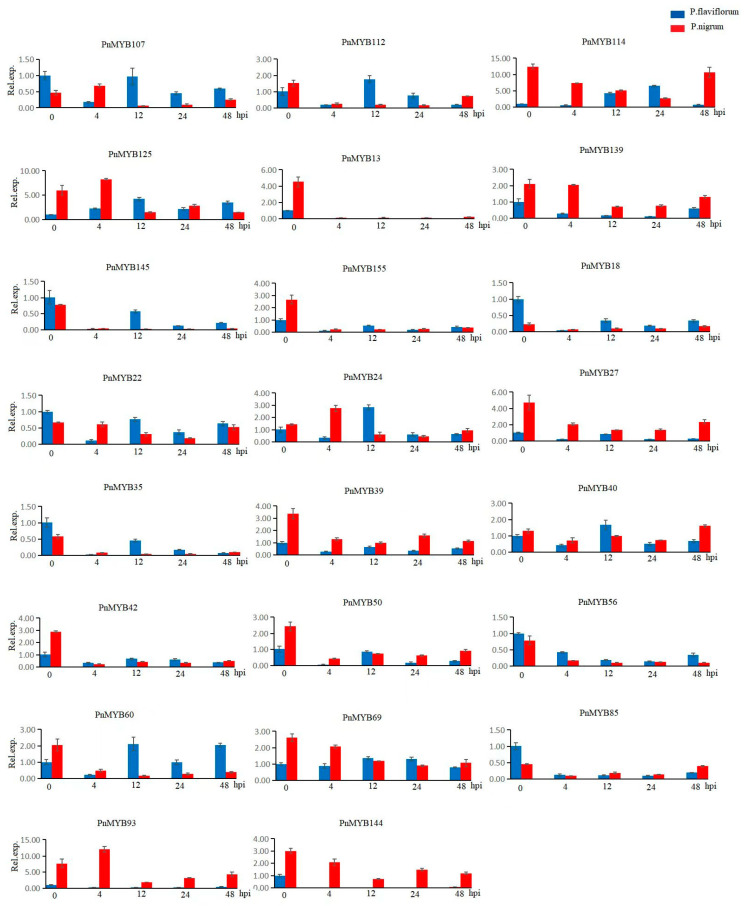
Expression profiling of 23 *PnMYB* genes at different times post *P. capsici* infection in *P. flaviflorum* and *P. nigrum*. *P. flaviflorum* and *P. nigrum* plants were treated with *P. capsici* for 0, 4, 12, 24, and 48 h for RNA isolation and qPCR. CK, control group not infected with *P. capsici*.

In order to gain deeper insight into the prospective roles of the various members of the *PnMYB* gene family, we examined the response of *PnMYB* gene family members in two species of *P. flaviflorum* and *P. nigrum* to *P. capsici* infection. RT-qPCR was employed to quantify the transcription levels of *PnMYB* gene members at various time points post infection. As illustrated in Figure 5, we obtained a total of 23 relative expression maps of *PnMYB* from 36 *PnMYB* samples of black pepper. After 4 h of *P. capsici* infection, *P. flaviflorum* and *P. nigrum* exhibited distinct *PnMYB* gene expression patterns compared to the control group, indicating the involvement of *PnMYB* genes in *P. capsici* transcription. In *P. flaviflorum*, the expression of 20 of 23 genes was downregulated after 4 h of infection with *P. capsici*, whereas the expression of *PnMYB125* was upregulated, and both *PnMYB114* and PnMYB69 maintained stable levels of expression. Similarly, in *P. nigrum*, the expression of 17 genes was downregulated, whereas 4 genes were upregulated, with the remainder exhibiting stable expression four hours post infection with *P. capsici*. Some genes presented fluctuating expression patterns as time progressed. For instance, at 12 h, the expression of the *PnMYB125* gene exhibited significant up-regulation in *P. nigrum* and notable down-regulation in *P. flaviflorum*. Furthermore, *PnMYB114* exhibited an up-regulation after 24 h in comparison to the control, with *P. nigrum* showing the most prominent up-regulation of expression in 24 h. For both Piper species, *PnMYB56* and *PnMYB85* indicated considerable down-regulation following infection as compared to the controls. In *P. nigrum*, the majority of *PnMYB* genes were downregulated at a specific time point (4 h after infection).

## 3. Discussion

Black pepper (*Piper nigrum* L.) is an important berry spice crop with high economic and medicinal value [4]. MYB is a large and multifunctional family of genes, and many gene families in plants belong to the MYB family [4]. The first MYB gene identified in plants was the maize C1 gene, which is involved in anthocyanin glycoside biosynthesis [36]. The plant-specific R2R3-MYB is the most abundant subfamily of the MYB family and plays an important role in plant responses to biotic and abiotic stresses. Even though members of the R2R3-MYB family have been identified in many species, the number of R2R3-MYBs varies across plant species, such as *A. thaliana* (138 members), *Oryza sativa* (155 members), and *Capsicum annuum* (108 members) [37,38]. In this study, we found that the black pepper genome contains 160 R2R3-MYB genes, which demonstrates the expanded abundance and pivotal role of this transcriptional family in black pepper. Chromosomal localization showed that 160 *PnMYBs* were randomly distributed across 26 chromosomes ranging in size from 0 to 50 Mb, each containing 1 to 11 genes.

Phylogenetic tree analysis, based on 160 PnMYB proteins and 125 AtMYB proteins, showed that R2R3-MYB family members of black pepper and *A. thaliana* clustered into 20 groups. The results of gene structure and motif analysis further supported this classification. The presence of members of *P. nigrum* and *A. thaliana* in each group suggests that *P. nigrum* and *A. thaliana* have a potential close relationship, possibly because they derive from a common ancestor and endure less variation in the course of evolution [39]. The distribution of *PnMYBs* in these 20 groups appeared to be uneven, with the largest group containing 17 *PnMYBs*. It tends to be conjectured that adaptive evolution might be more likely to occur within the larger subgroups of the phylogenetic tree [40].

Tandem duplication and segmental duplication constitute two primary mechanisms driving the origination and amplification of gene families in the genome [41]. The results of the collinearity analysis indicated the presence of gene duplication events in the black pepper genome, with the majority of the *PnMYB* genes among the 160 *PnMYBs* identified as segmentally duplicated, while four pairs of genes were tandemly duplicated. This suggests that the amplification of the MYB gene family in *P. nigrum* is mainly due to segmental duplications accompanied by tandem duplications. The expansion of transcription factor gene families in tobacco, camphor, potato, and pistacia chinensis was similar to this process [32,42]. In addition, nearly all (~98.97%) segmentally duplicated gene pairs demonstrated Ka/Ks ratios < 1, suggesting that most MYB genes in black pepper were driven by purifying selection. A study of mango MYB genes indicated some *MiMYBs* undergoing positive selection, suggesting a diversified natural selection process in play [33].

Diversity in gene structures and motifs largely influences gene family evolution, and the precise loss or acquisition of introns may be a vital factor contributing to the emergence of new genes [43]. Our study revealed an absence of introns in three genes, *PnMYB139*, *PnMYB133*, and *PnMYB134*, in Group 1, suggesting the occurrence of intron deletion in these genes. Similarly, 73.125% of the *PnMYBs* genes were found to have a typical splicing pattern of three exons and two introns. The diversity of these gene structures is essential for the evolution of black pepper and can provide the potential to foster novel functions in gene evolution and thereby facilitate improved adaptation to environmental changes [21]. Based on motif analysis, we identified 15 motifs in 160 *PnMYB* genes. Motif 3 and motif 4 were detected in all *PnMYB* genes. Within homologous clades, some motifs are unique and serve as the basis for gene family classification and functional differentiation, such as motif 11, motif 14, and motif 10. In black pepper, there is a high degree of similarity in both the type and number of motifs within the same subfamily, suggesting that the black pepper R2R3-MYB gene family is conserved and diverse. This result is line with previous studies in peanut, maize, pepper, and other species [38,44,45]. The nucleus was identified as the primary subcellular localization for most members of the black pepper *PnMYB* family. MYBs from many other species are also predicted to be nuclear proteins [21,25,46].

Foot rot, caused by *P. capsici*, is notably destructive to black pepper, severely impairing its yield and quality. Interestingly, *P. flaviflorum*, a rare wild pepper variant in China, has been shown to possess high resistance to pepper foot rot [47]. In this study, *P. nigrum* and *P. flaviflorum* were used as hosts exposed to *P. capsici* infection, and the differences in their response patterns in the MYB family were investigated at the transcriptomic level. The results showed that the expression of MYB family members in *P. nigrum* exhibited significant changes in both *P. nigrum* and *P. flaviflorum* after four cycles of *P. capsici* infection. Some of the genes divided in the same subgroup of the phylogenetic tree had closely similar expression profiles. Contrastingly, an opposite trend in expression patterns was seen between the different family system subgroups within *P. nigrum* and *P. flaviflorum*. This indicates that the gene function of the same subgroup has a high similarity. The expression patterns of the *PnMYBs* gene on *P. capsici* were very different between *P. nigrum* and *P. flaviflorum*. *PnMYB43* was highly expressed at only 4 h after *P. flaviflorum* infection with *P. capsici*. In another study, suppression of the expression of *BnMYB43* was shown to increase resistance to *S. sclerotinia* [48]. Insights into gene expression patterns are instrumental in comprehending gene functionality. In mango, the transcription of *MiMYB54* is regulated by MeJA and ROS, resulting in an immune response that enhances disease resistance [33]. Similarly, we speculated that after the infection of *P. capsici*, some PnMYB in pepper may also be regulated by some substances to enhance their expression ability. Of course, it may also be that *PnMYBs* itself has a better resistance to *P. capsica*. Previous studies indeed demonstrated that MYB might play a role in defense against *P. capsici* infection in other species. *CaMYB*-silenced leaves rendered more sporulation of *P. capsica,* indicating that *CaMYB* may play an important role in resistance to *P. capsici* in purple pepper [49].

In this study, most of the *PnMYBs* expression patterns changed significantly, and the expression patterns of *PnMYBs* changed slowly after 12 h of infection. These results indicated that most of the *PnMYBs* was induced to be expressed in both pepper plants after inoculation with *P. capsici* as detected by qPCR. Pepper plants infected with *P. capsici* exhibit symptoms such as xylem browning and black duct, which may be related to the regulation of lignin synthesis by the R2R3-MYB transcription factor [50]. Moreover, we observed a downregulated expression of 20 genes in *P. flaviflorum* plants 4 h after inoculation, while low expression levels of some MYB genes were observed in *P. nigrum* plants after inoculation. These results indicated that these genes showed distinct expression patterns in the defensive responses of *P. nigrum* and *P. flaviflorum*. The upregulated gene *MYB139* associated with late blight resistance in wild species was identified in *Solanum pinnatisectum* [51]. After *Sclerotinia sclerotiorum* inoculation, *BnMYB69* RNA interference plants showed a decrease in *S. sclerotiorum* resistance, implying that the *BnMYB69* gene family may be involved in the interaction with *S. sclerotiorum* in oilseed rape [52]. These genes have also been verified in our study, the results of which revealed that *PnMYBs* play a role in defending against *P. capsici*.

## 4. Materials and Methods

### 4.1. Plant Material

Healthy plants of black pepper were used for this study. The plants were grown under controlled conditions with a temperature of 25–27 °C, 85% relative humidity, and 8 h photoperiod. Five-node cuttings from one-year-old plants of *P. nigrum* and *P. flaviflorum* grown in the Piper species Germplasm Repository were taken for rooting. Those rooted cuttings were used as the experimental materials in this study. The pathogen *P. capsici* was incubated for seven days on potato dextrose agar plates. Immediately before inoculation, the midpoint of the third internode, as counted from the tip of a rooted cutting, was injured with a syringe needle. A 3 mm inoculating disk taken from the growing margin of *P. capsici* was then patched onto the injured point and covered with a wet cotton pad to prevent drying. The pad was tied onto the stem with a polyethylene strip to maintain the position of the inoculating disk. Inoculated plants were incubated at a constant temperature of 25–28 °C for 0, 4, 12, 24, and 48 h in a greenhouse with 75–90% relative humidity. Plants in the control group were injured in the same manner as described above, but instead of the agar disk, distilled water was applied to the injured point and then covered. Three root samples of each of the two species of Piper were collected at each of the five time points. All samples were frozen immediately in liquid nitrogen and later freeze-dried and stored at −80 °C until used for RNA-Seq, determination of chemical molecule contents, and qPCR. Three replicates of stem samples were also collected from the plants for lignin content determination.

### 4.2. Identification of MYB Genes in Pepper

A hidden Markov model was established based on known MYB protein sequences from 125 MYB family protein sequences obtained from the *Arabidopsis* Information Resource (http://www.arabidopsis.org/ (accessed on 30 September 2022)). This model was used to search for potential MYB family sequences in the coding protein sequences of black pepper. The BLASTP program (Version: ncbi-blast-2.10.1+) with default settings and an e-value of 1 × 10^−5^ was used to perform multiple sequence alignments of all black pepper protein sequences and the MYB family reference sequences, resulting in the identification of candidate MYB family sequences. The acquired candidate sequences were then domain-annotated using the pfamscan software (Version: v1.6) and the P famA database (Version: v33.1) to confirm the presence of the MYB domain. The physicochemical properties of the identified *PnMYBs*, such as the length of the amino acid sequence, molecular weight, theoretical isoelectric point, stability coefficient, hydrophilicity index, and index of lipid solubility, were analyzed using the online website ExPASy-ProtParam (http://web.expasy.org/protparam/ (accessed on 14 November 2022)).

### 4.3. Phylogenetic and Gene Structure Analysis

Multiple sequence alignments of black pepper MYB sequences were performed using Jalview (2.11.1.0) with default settings to identify sequence characteristics. To determine the evolutionary relationships and identify subfamilies, a phylogenetic tree was constructed using the neighbor-joining (NJ) method in MEGA (MEGA10) with MYB protein sequences from black pepper and *Arabipdopsis thaliana*. The resulting phylogenetic tree was visualized using ITOL-web (https://itol.embl.de/ (accessed on 27 February 2023)). The exon–intron organization of MYB genes was analyzed using the Gene Structure Display Serve (GSDS) tool (http://gsds.gao-lab.org/ (accessed on 13 March 2023)) based on information from the PnMYBs database. The identification of new motifs in PnMYBs was performed using MEME (Version: v5.0.5) with the following parameters: site distribution set to any number of repetitions (anr), number of motifs set to 15, and default values for other optional parameters.

### 4.4. Chromosomal Localization and Synteny Analysis

The chromosomal distribution of MYB genes in black pepper was visualized using Tbtools (1.047). Synteny analysis was conducted using MCScanX software (https://github.com/wyp1125/MCScanX (accessed on 19 August 2024)) with default parameters (MATCH_SCORE: 50; MATCH_SIZE: 5; GAP_PENALTY: −1; OVERLAP_WINDOW: 5; E_VALUE: 1 × 10^−5^; MAX_GAPS: 25).

### 4.5. Expression Profile Analysis of the MYB Gene Family in Black Pepper

The reference genome data utilized in this study were deposited in the NCBI Sequence Read Archive under accession number PRJNA529758 [53]. Expression Profile Analysis of the MYB Gene Family in black pepper was deposited in NCBI under accession numbers SRS5227911-SRS5227946. The data supporting the results of this study are included in Supplementary Table S1.

### 4.6. Quantitative Analysis of Candidate MYB Genes in Black Pepper

The expression levels of the MYB gene family in black pepper were analyzed using quantitative expression data. Total RNA was extracted from different tissues or different developmental stages. The integrity of the extracted RNA and DNA contamination were assessed using agarose gel electrophoresis and DNA digestion. Reverse transcription of total RNA was performed using the 4×Hifair III SuperMix Plus kit (Yesen, Shanghai, China). Real-time quantitative PCR with the SYBR GREEN dye technique was used to measure the relative expression levels of candidate MYB genes, with PUB1 as the internal reference gene. The relative expression of *PnMYB* genes was calculated using the 2^−∆∆ct^ method, and specific primers were designed with Primer5.0 (Supplementary Table S2).

## Data Availability

Data are contained within the article.

## Supplementary Materials

The following supporting information can be downloaded at: https://www.mdpi.com/article/10.3390/ijms25189851/s1.
