# Supplementary material for: Genome-Wide Identification, Characterization, and Expression Analysis of the MYB-R2R3 Gene Family in Black Pepper (Piper nigrum L.)
Source: Int J Mol Sci. 2024 Sep 12;25(18):9851. doi: 10.3390/ijms25189851 (PMC11432665; doi:10.3390/ijms25189851)
Supplement: Supplementary file 1 [file ijms-25-09851-s001.zip › ijms-3123543-supplementary figure legends.docx]

Figure S1. Multiple sequence alignment was performed for the MYB proteins in black pepper.

Figure S2. The number and length of *PnMYB* exons and introns vary.

Figure S3. Conserved motif analysis of *PnMYBs.*
