# Supplementary figures and images for: Genome-Wide Identification, Characterization, and Expression Analysis of the MYB-R2R3 Gene Family in Black Pepper (Piper nigrum L.)
Source: Int J Mol Sci. 2024 Sep 12;25(18):9851. doi: 10.3390/ijms25189851 (PMC11432665; doi:10.3390/ijms25189851)

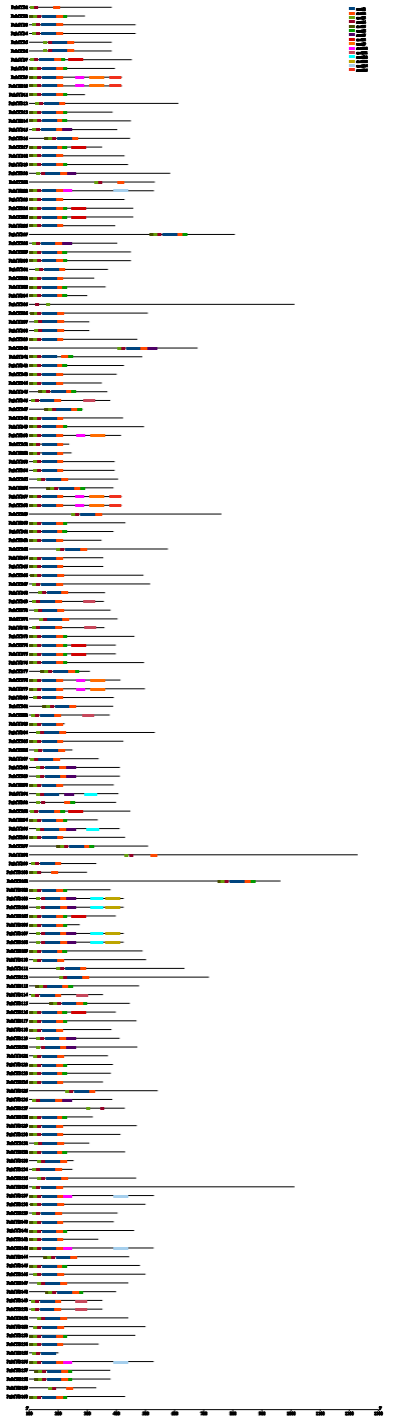

Supplement: Supplementary file 1 [file ijms-25-09851-s001.zip › Figure S3.pdf]

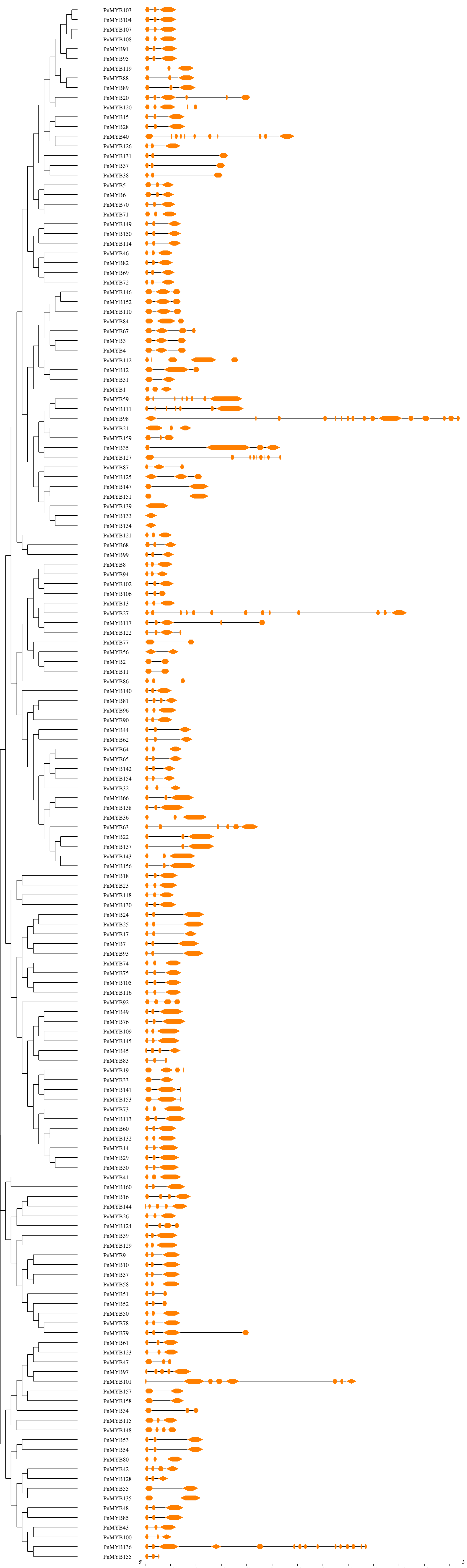

Supplement: Supplementary file 1 [file ijms-25-09851-s001.zip › Supplementary Figure S2.pdf]
